# Enhancement of Binding Affinity of Folate to Its Receptor by Peptide Conjugation

**DOI:** 10.3390/ijms20092152

**Published:** 2019-04-30

**Authors:** Roopa Dharmatti, Hideyuki Miyatake, Avanashiappan Nandakumar, Motoki Ueda, Kenya Kobayashi, Daisuke Kiga, Masayuki Yamamura, Yoshihiro Ito

**Affiliations:** 1 Nano Medical Engineering Laboratory, RIKEN Cluster for Pioneering Research, 2-1 Hirosawa, Wako, Saitama 351-0198, Japan; roopa.dharmatti@riken.jp (R.D.); motoki.ueda@riken.jp (M.U.); kenya.kobayashi@alpsalpine.com (K.K.); 2 Department of Computer Science, School of Computing, Tokyo Institute of Technology, 4259 Nagatsuta-cho, Midori-ku, Yokohama 226-8503, Japan; kiga@waseda.jp (D.K.); my@c.titech.ac.jp (M.Y.); 3 Emergent Bioengineering Materials Research Team, RIKEN Center for Emergent Matter Science, 2-1 Hirosawa, Wako, Saitama 351-0198, Japan; nandakumar.avanashiappan@riken.jp; 4 Department of Electrical Engineering and Bioscience, Waseda University, 2-2 Wakamatsu Cho, Shinjyuku-ku, Tokyo 162-8480, Japan

**Keywords:** folate, folate receptor, peptide conjugation, click reaction, biolayer interferometry

## Abstract

(1) Background: The folate receptor (FR) is a target for cancer treatment and detection. Expression of the FR is restricted in normal cells but overexpressed in many types of tumors. Folate was conjugated with peptides for enhancing binding affinity to the FR. (2) Materials and Methods: For conjugation, folate was coupled with propargyl or dibenzocyclooctyne, and 4-azidophenylalanine was introduced in peptides for “click” reactions. We measured binding kinetics including the rate constants of association (*k_a_*) and dissociation (*k_d_*) of folate-peptide conjugates with purified FR by biolayer interferometry. After optimization of the conditions for the click reaction, we successfully conjugated folate with designed peptides. (3) Results: The binding affinity, indicated by the equilibrium dissociation constant (*K_D_*), of folate toward the FR was enhanced by peptide conjugation. The enhanced FR binding affinity by peptide conjugation is a result of an increase in the number of interaction sites. (4) Conclusion: Such peptide-ligand conjugates will be important in the design of ligands with higher affinity. These high affinity ligands can be useful for targeted drug delivery system.

## 1. Introduction

Traditional cancer therapy involves removal of tumor cells by surgery, radiation and non-selective types of chemotherapy [1,2]. Surgery and radiation are often effective with tumors that are primary or localized and have not metastasized to multiple sites throughout the body [3]. Chemotherapy is effective in the treatment of metastatic cancers because typical chemotherapeutic agents focus on rapidly growing tissues, which is a property common to cancer cells. Nonetheless, chemotherapy also often has a high incidence of unwanted and damaging side effects in normal tissues because these tissues are also undergoing growth [4,5]. Therefore, monoclonal antibodies against cellular targets that are unique to cancer cells have been developed [4,6], and antibody-drug conjugates (ADCs) have also been developed [6]. Targeted treatments exert their anticancer effects through multiple mechanisms, including proliferation inhibition [6], apoptosis induction [7], metastasis suppression [8], immune function regulation [9] and multidrug resistance reversal [5,10]. A few ADCs have been used successfully in clinical trials [5,10,11]. However, there are several points to consider when using an antibody as a drug-transporter that targets tumors. Limitations owing to poor therapeutic efficacy of ADCs include: (i) manufacturing procedures that create heterogeneous mixtures of ADCs with a number of drug molecules conjugated inconsistently; (ii) the synthesis costs are extremely high with difficulties in quality control; and (iii) the larger size of ADCs hampers penetration of ADCs into tumor tissue [12]. Small molecules or peptides are potential therapeutic molecules that overcome these problems [2]. In contrast to antibodies, these agents provide advantages such as reduced immunogenicity, quick clearance, increased diffusion and tissue penetration, chemical stability and ease of synthesis [2,6].

Due to the remarkable expression of the folate receptor (FR) on the surface of tumor cells, the FR can be exploited as a cancer diagnostic and therapeutic target [13]. Folate is an intrinsic ligand of the FR, consisting of a pterin ring, a central *p*-amino benzoic acid and an L-glutamic acid tail [4,14,15], and has been conjugated with anti-cancer drugs [4,16] and drug carriers [17,18,19,20,21,22,23] for targeted delivery of drugs to tumor cells. For example, a peptide that binds to the α isoform of the FR, which is a subtype of FRs, was selected by phage display; however, the affinity of this peptide was low when compared with that of folate [24].

**Figure 1:**
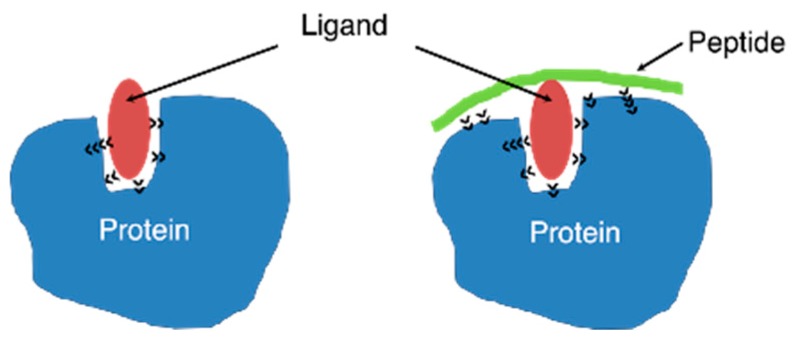
Schematic showing how a peptide conjugated to a ligand (folate) enhances the affinity of the ligand toward the target protein (folate receptor). The black arrowheads indicate molecular interactions.

In this report, we conjugate folate with peptides to enhance binding affinity toward the FR. Previously, Li and Roberts [25] prepared a penicillin-peptide conjugate that has at least 100-fold higher activity than penicillin. Wang et al. introduced aminophenylalanine coupled with purvalanol into peptides to enhance the inhibitory activity of purvalanol against kinases [26,27]. Peptide conjugation should increase the affinity between the target protein and ligand by increasing the number of interaction sites, as shown in Figure 1.

**Figure 2:**
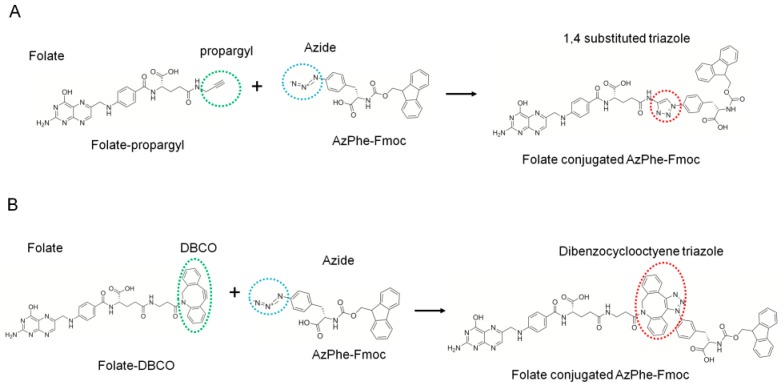
Schemes showing the synthesis of the folate-conjugated AzPhe-Fmoc. (**A**) The Cu(I)-catalyzed alkyne-azide cycloaddition (CuAAC) click reaction between the propargyl group (green dotted circle) and the azide group (cyan dotted circle) to conjugate folate via the triazole ring (red dotted circle). (**B**) The strain-promoted alkyne-azide cycloaddition (SPAAC) click reaction between DBCO (green dotted circle) and the azide group (cyan dotted circle) to conjugate folate via dibenzocyclooctyne triazole (red dotted circle).

For conjugation, we added propargyl or dibenzocyclooctyne (DBCO) to folate and 4-azidophenyalanine (AzPhe) in the peptide for the “click” reaction, as shown in Figure 2, because it is possible to introduce the azidophenyl groups into proteins by bio-orthogonal approaches [28,29]. The binding assay of the synthesized folate-peptide conjugates with FR was performed by biolayer interferometry (BLI), and the association rate constant (*k*_a_) and dissociation rate constant (*k*_d_) were determined. The study demonstrated that the conjugation of folate with peptides enhanced the affinity of folate toward the FR.

## 2. Results and Discussion

### 2.1. Folate-Phe Conjugation by Click Reactions

Two types of folate analogues were prepared by addition of the propargyl group (Figure 2A) and DBCO (Figure 2B), and both were adjacent to the γ-carboxyl group of folate. The additions enabled confirmation of the click reaction between folate analogues and AzPhe-Fmoc. Folate-propargyl was used for the Cu(I)-catalyzed alkyne-azide cycloaddition (CuAAC) click reaction with AzPhe-Fmoc. To promote the CuAAC reaction, Cu(I) stabilizing ligands such as Tris (2-benzimidazoylmethyl) amine (BimH_3_) and microwaves were also employed at 50 °C. However, absorbance from the triazole ring on the target compound was not detected under the conditions shown in Table 1.

Currently, some groups have reported success of the CuAAC reaction between folate-propargyl and polymers containing an azido group [30,31,32,33,34]. However, their folate-propargyl conjugates were a mixture of propargyl groups bound to the C^α^ and C^γ^ of the glutamic acid part of folate. The present conjugate is the first example of a folate-propargyl with the propargyl group specifically linked to the C^γ^ of folate. The results in Table 1 indicate that the C^γ^-binding propargyl group shows low reactivity in the CuAAC reaction. The other possibility is that coordination by the -N and -NH groups of the folate-propargyl with Cu(I) interferes with alkyne-Cu(I) complexation.

In contrast, the strain-promoted azide-alkyne cycloaddition (SPAAC) “click” reaction between folate-DBCO and AzPhe-Fmoc was successful (Table 1). The yield increased up to 88% by using twice the molar ratio of folate-DBCO against AzPhe-Fmoc, and the reaction temperature did not affect yields noticeably. Golas et al. [35] studied the substituent effect on azide reactivity in CuAAC using various azide compounds with propargyl alcohol. The electronic properties and steric congestion near end groups are major determinants for the reactivity of azide compounds. Azide with electron withdrawing groups, such as ethyl azido-acetate, methyl 2-azidopropionate and azidoacetonitrile, react faster than similar compounds with a neighboring aromatic ring (benzyl azide and 1-phenylethyl azide). In addition, primary azides such as benzyl azide and ethyl azido-acetate react faster than their secondary analogues, 1-phenylethyl azide and methyl 2-azidopropionate, respectively. In this case, AzPhe is less reactive because the electron-withdrawing is affected by the aromatic ring. Nonetheless, AzPhe can be more reactive through SPAAC because DBCO enhances the reactivity by its resonant structure [36].

### 2.2. Preparation of Folate-Peptide Conjugates by the SPAAC Click Reaction

**Figure 3:**
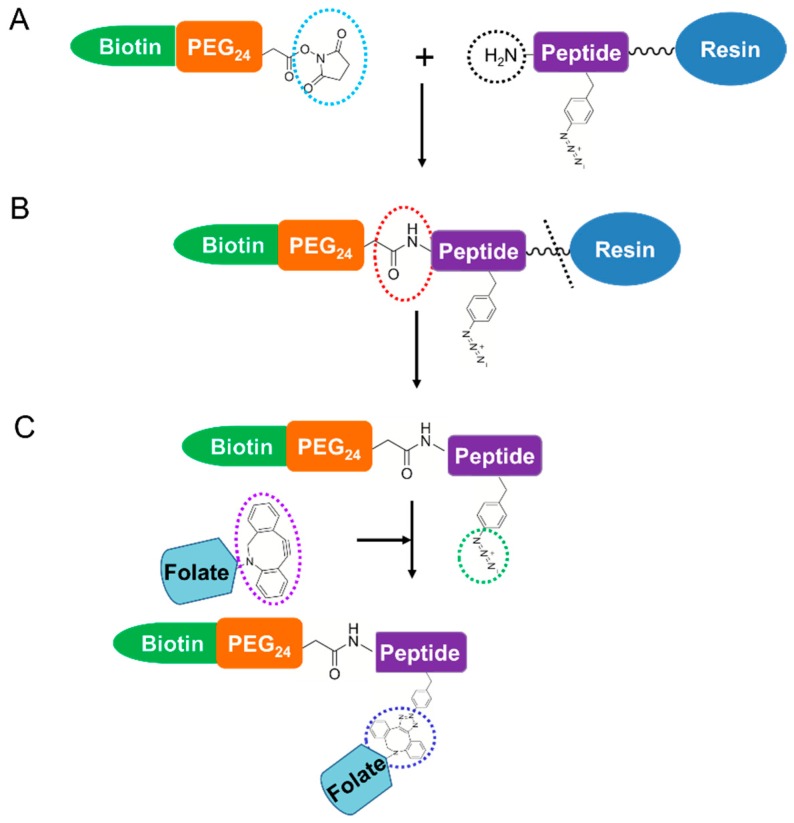
Synthesis procedure for peptide conjugates. (**A**) *N*-terminal peptide modification with biotin-PEG_24_ was achieved by reacting ester NHS (cyan dotted circle) and the NH_2_ group of the peptide (black dotted circle).The black wavy line between resin and peptide indicated various peptide lengths. (**B**) After the *N*-terminal modification with biotin-PEG_24,_ the amide bond (red dotted circle) was formed. Next, peptide was cleaved from resin (black dotted line). (**C**) The folate-DBCO-AzPhe containing peptide was achieved by the SPAAC click reaction between DBCO (purple dotted circle) of the folate and azide groups (green dotted circle) of the AzPhe in the peptide to form the folate-peptide conjugate via the dibenzocyclooctyne triazole (blue dotted circle).

Since folate-DBCO was demonstrated to conjugate efficiently to AzPhe by the SPAAC reaction, the preparation of folate-peptide conjugates was performed by this reaction (Figure 3). Three peptide sequences, GF[AzPhe]IQ, SE[AzPhe]KA and DSE[AzPhe]KAY, were synthesized. The folate-peptide conjugates were designed by the program ICM-Pro (Molsoft L.L.C., San Diego, CA, USA). After successful conjugation of folate with AzPhe by SPAAC, we considered the folate-conjugated AzPhe as one unit and increased the length of the peptide by adding amino acids at N-terminal and C-terminal of the AzPhe. This length was increased by trial and error procedure. The peptides were synthesized by a conventional solid phase synthesis method. For BLI measurements, in which a biotin group binds to streptavidin bound to coated sensor chips, the N-terminus of the peptides was modified with biotin-(PEG_24_)-NHS. The coupling was performed before release from the solid phase synthesis resin (Figure 3A) [37,38]. The polyethylene glycol (PEG) linker functions as a spacer between the immobilized and interaction sites and as a solubilizer of the folate-peptide conjugates in aqueous solutions. 

The same coupling reaction conditions were used for peptide conjugation. After the click reaction and purification, each folate-peptide conjugate was identified by matrix assisted laser desorption/ionization-time of flight mass spectrometry (MALDI-TOF MS). From the mass spectra, folate was confirmed to bind successfully to the side chain of AzPhe in the peptides.

### 2.3. BLI Measurement

**Figure 4:**
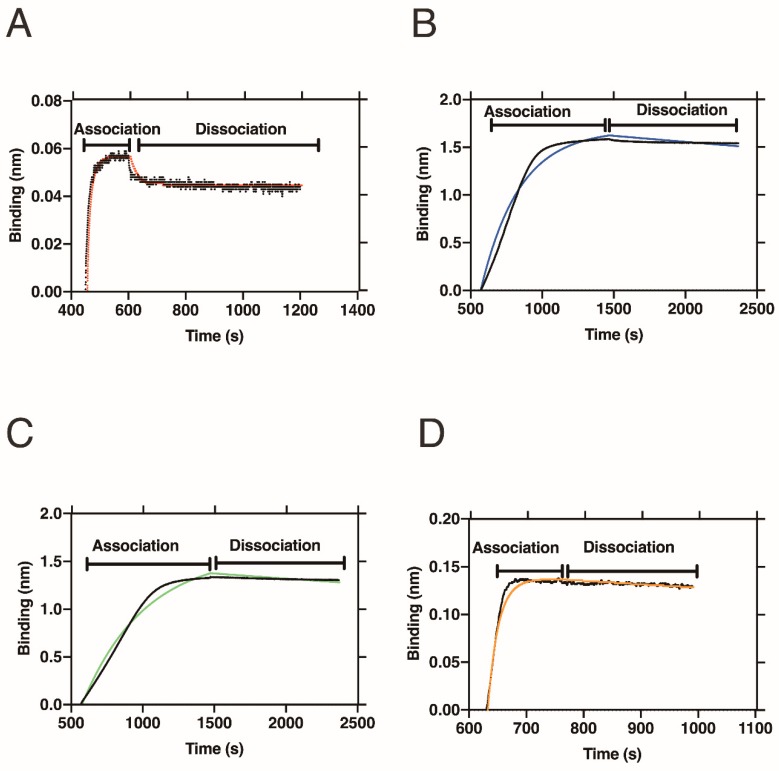
BLI data for binding of (**A**) folate-PEG_8_-biotin, (**B**) GF**Z**IQ, (**C**) SE**Z**KA and (**D**) DSE**Z**KAY with FRα. In all cases, analyte only data was kept as a reference and 1:1 local analysis was used. The black curve lines are run data and colored curve lines are fitting data.

Table 2 and Figure 4 show the results of the BLI measurements to evaluate the affinities of the folate-peptide conjugates toward folate receptor alpha (FRα). 

Commercially available folate-PEG_8_-biotin was used as a control for BLI analysis. The equilibrium dissociation constant (*K*_D_) between FRα and folate was 1.14 nM. Wibowo et al. [39] and Chen et al. [14] used a radiolabeled ligand assay and isothermal calorimetry for measurement of the *K*_D_ of folate with FRα and yielded values of ~10 pM and ~190 pM, respectively. Combined with our results, the differences in *K*_D_ values indicate that the method used to measure the *K*_D_ has a strong influence on the outcome.

An advantage of BLI is evaluation of the *k*_a_ and *k*_d_. The binding mode of folate to FRα shows a non-equilibrium binding mode, in which the *k*_d_ (7.69 × 10^−3^ s^−1^) was ~10^3^ times slower than that of the association rate (6.74 × 10^6^ M^−1^ s^−1^). This difference between the *k*_a_ and *k*_d_ corresponds well with the scenario previously proposed for folate binding to FRα [39]. In crystallographic work that compared the apo- and folate binding forms of FRs, large conformational changes around the folate binding pocket upon folate binding were observed, i.e., from the relaxed (open) to tight (closed) forms. In the closed form, the inhibitory loop, basic loop and switching helix around the binding pocket cooperatively undergo conformational changes to bind the folate tightly. The bound folate in the FRs then dissociates from the receptors after endocytosis of the FRs into cells, which is triggered by the acidic environment of the cells. Such a non-equilibrium-binding mode promotes efficient uptake of folate into cells. Thus, our BLI data provide the first indication that the proposed trafficking mechanism of folate is valid by revealing the asymmetric binding kinetics of FRs.

### 2.4. Interaction of Folate-Peptide Conjugates with FRα

By conjugation with peptides, the affinity increased to sub-nanomolar (~10^−10^ M) *K*_D_ values (Table 2). The peptide-conjugates showed slower *k*_a_ values that ranged from 8.91 × 10^4^ to 1.10 × 10^6^ (M^−1^ s^−1^). Results presented in Figure 4B–D show significantly slow dissociation even after incubation in buffer. As a result, the *k*_d_ slows from 7.53 × 10^−5^ to 2.65 × 10^−4^ s^−1^, which increases the *K*_D_ values. These observations suggest that peptide modification further stabilizes the complex formed between the peptide-conjugates and FRs, most probably by increasing the number of interaction sites between them. 

In the peptide-conjugates, SE**Z**KA and DSE**Z**KAY share the common SE**Z**KA sequence. Addition of aspartic acid (D) at the N-terminus and tyrosine (Y) at the C-terminus leads to a 12-fold faster association constant and 3-fold faster dissociation constant for the DSE**Z**KAY peptide-conjugate, resulting in a 4-fold lower *K*_D_. This increase in affinity occurs by lengthening SE**Z**KA to DSE**Z**KAY. This result indicates that we can alter the affinity of peptide-conjugate compounds by increasing the length of the peptides at both the N- and C-termini. This may provide a way to manipulate binding properties of peptide-conjugated compounds by increasing the length of the peptide part of the conjugates, which may increase the number of interaction contacts with the target protein. 

**Figure 5:**
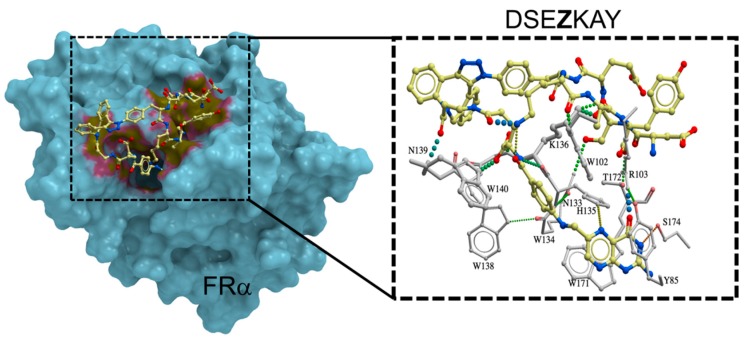
Docking model of DSE**Z**KAY (yellow) with the surface of the FRα (blue). The gradation of yellow and magenta colors on the surface of the FRα indicated the interaction between the ligand and surface of the FRα. This figure was prepared by the program ICM-Pro. The left dotted box area of interaction between DSE**Z**KAY with FRα is zoomed in right dotted box. All the dotted lines in right dotted box indicate an “increased” interaction of DSE**Z**KAY with the FRα (except for the interaction of folate with the FRα).

Figure 5 shows the results of the docking simulation, which demonstrates the interaction mode of DSE**Z**KAY with FRα. As expected in Figure 1, the structure of the complex shows an increase in the number of interactions to FRα from the peptide portion around the folate-binding pocket. Previous reports have demonstrated greater than 100-fold increases in binding affinity by peptide conjugates [25,26,27], whereas the present result was lower than these previous increases in affinity. However, the present investigation also revealed that peptide conjugation is a useful tool to enhance the binding affinity to the target molecule. Future efforts will focus on using the folate-peptide conjugate to target anti-cancer drug delivery.

## 3. Materials and Methods

### 3.1. Materials

Fmoc-Phe(4-N_3_)-OH (AzPhe-Fmoc) was purchased from Watanabe Chemical Industries, Ltd. (Hiroshima, Japan) to incorporate non-natural amino acids during the solid phase peptide synthesis procedure. BimH_3_ was purchased from Tokyo Chemical Industry Co., Ltd. (Tokyo, Japan). Biotin-PEG_24_-NHS was purchased from Thermo Fisher Scientific (Waltham, MA, USA) for biotin-PEG_24_ modification at the N-terminus of the folate-peptide conjugates. For the activity assay, streptavidin (SA) biosensors were purchased from ForteBio (Fremont, CA, USA). Folate-PEG_8_-biotin was purchased from Nanocs (New York, NY, USA). Reagents used for reversed-phase high performance liquid chromatography (RP-HPLC) were of HPLC grade. All other chemicals used were of biochemical research grade. MALDI-TOF MS (Microflex, Bruker Daltonics, Billerica, MA, USA.) was employed for molecular weight measurement.

### 3.2. Synthesis of Folate-Propargyl and Folate-DBCO

**Figure 6:**
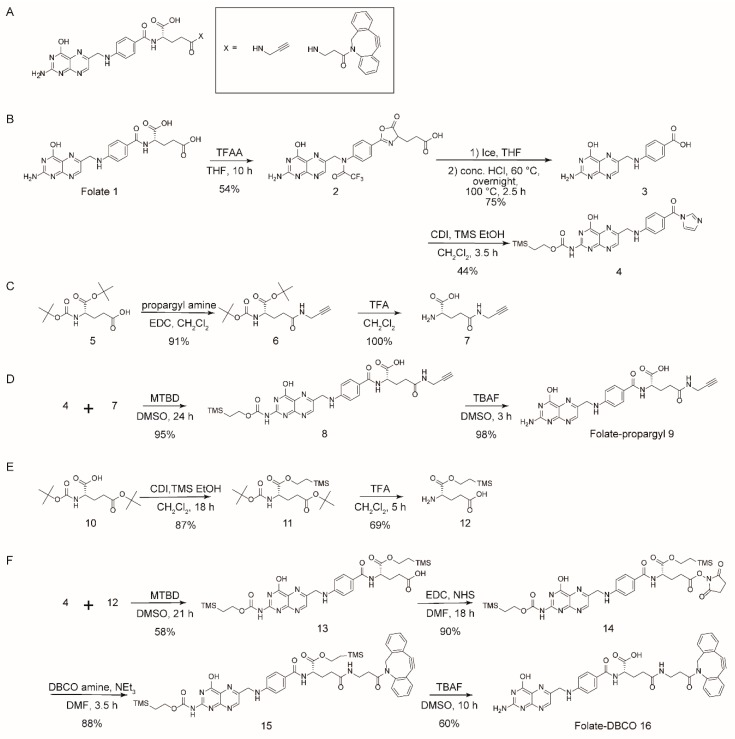
Structures and syntheses of folate derivatives. (**A**) Structure of folate is modified with group ‘X’, where X = propargyl or DBCO (the chemical structures was drawn in black box). The chemical structures of propargyl and DBCO are drawn in black box. (**B**) Synthesis of pteroic acid, (**C**) synthesis of γ-propargyl glutamic acid, (**D**) synthesis of folate-propargyl, (**E**) synthesis of silyl protected glutamic acid and (**F**) synthesis of folate-DBCO.

Synthetic schemes of folate derivatives are presented in Figure 6. Each compound was synthesized and confirmed as follows.

#### 3.2.1. Compound **2**

To a solution of folate **1** (10 g, 0.022 mol) and 100 mL anhydrous tetrahydrofuran (THF) in a three-neck flask, 24 mL, 0.176 mol trifluoroacetic anhydride [(CF_3_CO)_2_O] was slowly added at 0 °C for 30 min. The dark brown homogeneous mixture was stirred at room temperature (RT). After 10 h, the reaction mixture was filtered through a pad of celite to remove the small amount of solid residue. The filtrate was concentrated under reduced pressure and the viscous liquid was dissolved with a minimum amount of THF (5 mL), which was slowly transformed into a flask of well-stirred diethyl ether (Et_2_O). The yellow precipitate formed in Et_2_O was collected by filtration and washed with Et_2_O (25 mL ×2) to yield the crude compound **2**.

#### 3.2.2. Compound **3**

The crude compound **2** (6 g) was dissolved in THF (50 mL) followed by the addition of ice (~10 g) with stirring for 5 h. The mixture was slowly transferred into stirred Et_2_O (200 mL). The yellowish precipitate was collected by filtration, washed with Et_2_O (200 mL ×3) and dried for 24 h under vacuum. To the suspension of yellowish precipitate, conc. HCl (60 mL) was added and refluxed at 60 °C overnight and then 100 °C for 2.5 h. The reaction mixture was poured into water (100 mL). The precipitate formed in the solution was collected by filtration and washed with Et_2_O to afford compound **3** (75%). ^1^H NMR (DMSO-d_6_, 400 MHz): 4.60 [singlet (s), 2 H], 6.66 [doublet (d), *J* = 8.8 Hz, 2 H], 7.66 (d, *J* = 8.8 Hz, 2 H), 8.68 [broad singlet (brs), 2 H), 8.78 (s, 1 H).

#### 3.2.3. Compound **4**

Compound **3** (3.0 g, 9.6 mmol), Et_3_N (5.36 mL, 38.0 mmol), and 1,1′-carbonyldiimidazole (CDI) (6.2 g, 38.0 mmol) in 30 mL dimethyl sulfoxide (DMSO) was stirred at RT for 3.5 h. To the resulting solution, 2-(trimethylsilyl) ethanol (11 mL, 76.8 mmol) was added. After 5 h stirring at RT, the reaction mixture was poured into a mixture of water (330 mL), 9.6 mL acetic acid (AcOH) and Et_2_O (192 mL). The resulting yellow precipitate was collected by filtration and purified on a silica gel column with 10% (*v*/*v*) methanol (MeOH) in CHCl_3_ to give a yellow solid, which was further washed with Et_2_O to give compound **4** (2.14 g, 44%). ^1^H NMR (DMSO-d_6_, 400 MHz): 0.06 (s, 9H), 1.03–1.07 [multiplet (m), 2H], 4.28–4.32 (m, 2H), 4.66 (d, *J* = 6.4 Hz, 2H), 6.79 (d, *J* = 8.8 Hz, 2H), 7.10 (s, 1H), 7.61–7.67 (m, 4H), 8.15 (s, 1H), 8.89 (s, 1H). 

#### 3.2.4. Compound **6**

To compound **5** (800 mg, 2.6 mmol) in THF (20 mL), propargylamine hydrochloride (275 mg, 3.0 mmol), 1-ethyl-3-(3-dimethylaminopropyl) carbodiimide hydrochloride (EDC) (575 mg, 3.0 mmol) and Et_3_N (575 mL) were added, and subsequently CH_2_Cl_2_ (30 mL) and MeOH (10 mL) were added to dissolve solids completely. After stirring at RT for 5 h the solvent was evaporated. The residue was purified on a silica gel column to give compound **6** (620 mg, 91%). ^1^H NMR (CDCl_3_, 400 MHz): 1.46 (s, 18 H), 1.86 (s, CC*H*, 1 H), 2.12–2.31 (m, 4 H), 4.06 [quartet (q), 2 H), 4.16 (m, ^α^C*H*, 1 H), 5.24 (s, N*H*, 1 H), 6.52 (s, N*H*, 1 H).

#### 3.2.5. Compound **7**

Compound **6** (620 mg, 1.8 mmol) was dissolved in CH_2_Cl_2_ (5.4 mL) and cooled to 0 °C. To the solution, 12.6 mL trifluoroacetic acid (TFA) was added while stirring. After stirring at RT for 4 h, the solvent was evaporated under reduced vacuum. MeOH was added to dissolve the crude powder and then solidification was performed by the addition of Et_2_O. The solvent was evaporated and the precipitate dried to give compound **7** (340 mg, 100%). ^1^H NMR (D_2_O, 400 MHz): 2.00 (dd, 2 H), 2.30 (dd, 2 H), 2.45 (s, CC*H*, 1 H), 3.66 (t, ^α^C*H*, 1 H), 3.81 (s, 2 H).

#### 3.2.6. Compound **8**

Compound **7** (107 mg, 0.58 mmol), compound **4** (224 mg, 0.44 mmol) and 7-methyl-1,5,7-triazabicyclo [4.4.0] dec-5-ene (MTBD) (0.2 mL) were dissolved in DMSO (5 mL) and the mixture was stirred for 24 h under a nitrogen atmosphere. The solution was drop-wise added into a mixture of 1 M AcOH (30 mL), MeOH (15 mL) and CHCl_3_ (30 mL). The solution was then washed with a AcOH:MeOH (1:1, *v*/*v*) mixture once and with a H_2_O:MeOH (2:1, *v*/*v*) mixture twice. The organic layer was then dried with MgSO_4_ and evaporated. The solid was dissolved in a minimum volume of CHCl_3_ and was solidified by the addition of Et_2_O. The precipitate was collected by decantation and the solvent evaporated, and the precipitate dried under vacuum to yield compound **8** (263 mg, 95%). ^1^H NMR (DMSO-d_6_, 400 MHz): 0.05 (s, 9 H), 1.05 (m, 2 H), 1.91 (m, 2 H), 2.20 (m, 2 H), 3.07 (s, CC*H,* 1 H), 3.83 (s, 2 H), 4.30 (m, 3 H), 4.60 (d, 2 H), 6.66 (d, 2 H), 7.03 [triplet (t), folate amine, 1 H), 7.65 (d, 2 H), 8.17 (br, folate amide, 1 H), 8.30 (br, amide, folate amide, 2 H), 8.84 (s, 1 H), 11.9 (br, folate O*H*, 2 H). MALDI-TOF MS calculated for C_28_H_34_N_8_O_7_Si [M + H]^+^ 623.239; obtained [M+H]^+^ 623.403.

#### 3.2.7. Compound **9** (Folate-Propargyl)

To a solution of compound **8** (70 mg, 0.11 mmol) in DMSO (1 mL), 1 M tetrabutylammonium fluoride (TBAF) in THF (1 mL) was added and then stirred. After 3 h stirring at RT, the mixture was solidified by the addition of H_2_O:AcOH (2:1, *v*/*v*), and the material purified by centrifugation and decantation. This procedure was performed three times. The compound was solidified by Et_2_O and centrifuged once. The orange powder of compound **9** (53 mg, 98%) was obtained by drying *in vacuo.* Figure S1 shows ^1^H NMR data of compound **9**. ^1^H NMR (DMSO-d_6_, 400 MHz): 1.94 (m, 2 H), 2.21 (t, 2 H), 3.07 (s, CC*H*, 1 H), 3.82 (s, 2 H), 4.26 (m, 1 H), 4.48 (d, 2 H), 6.64 (d, 2 H), 6.93 (t, folate amine, 1 H), 7.65 (d, 2 H), 8.13 (d, folate amide, 1 H), 8.29 (t, folate amide, 1 H), 8.65 (s, 1 H), 12.0 (br, folate O*H*, 2 H). MALDI-TOF MS calculated for C_22_H_23_N_8_O_5_ [M + H]^+^ 479.179; obtained [M + H]^+^ 479.310.

#### 3.2.8. Compound **11**

A mixture of compound **10** (3 g, 9.9 mmol) and CDI (1.60 g, 9.9 mmol) in CH_2_Cl_2_ (30 mL) was stirred at RT for 1 h, followed by the addition of 1.46 mL of 9.9 mmol tetramethylsilane ethanol (TMS EtOH), and this sample was stirred for a further 18 h. H_2_O (150 mL) was added to the reaction mixture and the resulting mixture was partitioned. The organic layer was dried with anhydrous Na_2_SO_4_ and the solvent evaporated under reduced pressure. The residue was purified on a silica gel column with 25% (*v*/*v*) ethyl acetate in hexane to give a colorless oil 11 (3.46 g, 87%). ^1^H NMR (DMSO-d_6_, 400 MHz): 0.05 (s, 9 H), 0.99–1.04 (m, 2 H), 1.44–1.45 (m, 18 H), 1.85–1.95 (m, 1 H), 2.07–2.16 (m, 1 H), 2.24–2.38 (m, 2 H), 4.20–4.30 (m, 3 H), 5.09 (d, *J* = 8.4 Hz, 1 H).

#### 3.2.9. Compound **12**

A mixture of compound **11** (2 g, 4.9 mmol) and TFA:CH_2_Cl_2_ (1:2, *v*/*v*) (15 mL) was stirred at 0 °C for 30 min. The reaction mixture was then allowed to acquire at RT for 4.5 h. while stirring. The solvent of the reaction mixture was evaporated and the material purified on a silica gel column with 20–35% (*v*/*v*) MeOH in CHCl_3_ to give compound **12** (0.842 g, 69%, as a colorless semisolid). ^1^H NMR (DMSO-d_6_, 400 MHz): 0.05 (s, 9 H), 0.99–1.03 (m, 2 H), 1.94–2.05 (m, 2 H), 2.36–2.48 (m, 2 H), 4.03 (t, *J* = 6.4 Hz, 1 H), 4.22–4.26 (m, 2 H).

#### 3.2.10. Compound **13**

Compound **12** (1.75 g, 3.4 mmol), compound **4** (1.28 g, 5.2 mmol) and MTBD (1.48 mL, 10 mmol) in DMSO (15 mL) were stirred at RT in a 100 mL two neck round bottom flask. After 21 h, the resulting mixture was poured into a mixture of aqueous AcOH (1 M, 600 mL), MeOH (250 mL) and CHCl_3_ (600 mL). The organic layer was then washed with 1 M AcOH:MeOH (1/1, *v*/*v*) (400 mL) and H_2_O:MeOH (2/1) (600 mL ×2). The resulting organic solution was dried with anhydrous Na_2_SO_4_ and evaporated under reduced pressure. The crude mixture was purified on a silica gel column with CHCl_3_:MeOH:ethyl acetate:AcOH (17:1:2:0.08, *v*/*v*/*v*/*v*) and then CHCl_3_:MeOH:AcOH (9:1:0.025, *v*/*v*/v) to afford a yellow solid compound **13** (1.38 g, 58%). ^1^H NMR (DMSO-d_6_, 400 MHz): 0.01 (s, 9 H), 0.06 (s, 9 H), 0.91–0.95 (m, 2 H), 1.03–1.07 (m, 2 H), 1.86–1.95 (m, 1 H), 1.99–2.06 (m, 1 H), 2.30–2.34 (m, 2 H), 4.09–4.13 (m, 2 H), 4.28–4.34 (m, 3 H), 4.59 (d, *J* = 6 Hz, 2 H), 6.65 (d, *J* = 8.8 Hz, 2 H), 7.02 (t, *J* = 6.4 Hz, 1 H), 7.65 (d, *J* = 8.4 Hz, 2 H), 8.22 (d, *J* = 7.6 Hz, 1 H), 8.84 (s, 1 H), 11.88 (br, 4 H).

#### 3.2.11. Compound **14**

To a solution of compound **13** (1 g, 1.5 mmol) and 5 mL *N*,*N*-dimethylformamide (DMF), NHS (202 mg, 1.7 mmol) and EDC (279 mg, 1.5 mmol) were added. The resulting mixture was stirred at RT for 18 h. The reaction mixture was poured into water (300 mL) and the yellow precipitate was collected by filtration to afford compound **14** (1.03 g, 90%). ^1^H NMR (DMSO-d_6_, 400 MHz): 0.00 (s, 9 H), 0.06 (s, 9 H), 0.91–0.95 (m, 2 H), 1.02–1.07 (m, 2 H), 2.04–2.16 (m, 2 H), 2.76–2.84 (m, 6 H), 4.09–4.15 (m, 2 H), 4.28–4.32 (m, 3 H), 4.59 (s, 2 H), 6.66 (d, *J* = 8.8 Hz, 2 H), 7.66 (d, *J* = 9.2 Hz, 2 H), 8.32 (d, *J* = 7.6 Hz, 1 H), 8.84 (s, 1 H), 11.70 (br, 2 H).

#### 3.2.12. Compound **15**

Compound **14** (142 mg, 0.18 mmol), DBCO amine (50 mg, 0.18 mmol) and triethylamine (Et_3_N) (0.04 mL, 0.29 mmol) in 3 mL CH_2_Cl_2_ were added and stirred at RT for 3.5 h in a 20 mL round bottom flask. The reaction mixture was diluted with CHCl_3_ (25 mL) and washed with water (25 mL ×2). The organic layer was dried with anhydrous Na_2_SO_4_, evaporated and the sample purified on a Sephadex LH-20 column with CHCl_3_:MeOH = 1:1 (*v*/*v*) to afford compound **15** (0.150 mg; 88%). ^1^H NMR (DMSO-d_6_, 400 MHz): −0.007 (s, 9H), 0.054 (s, 9 H), 0.89–0.93 (m, 2 H), 1.02–1.06 (m, 2 H), 1.75–2.08 (m, 5 H), 2.36–2.43 (m, 1 H), 2.90–2.95 (m, 1 H), 3.04–3.12 (m, 1 H), 3.60 (dd, *J* = 13.6 Hz, *J* = 6.8 Hz, 1 H), 4.07–4.11 (m, 2 H), 4.21–4.31 (m, 3 H), 4.59 (d, *J* = 5.6 Hz, 2 H), 5.01 (t, *J* = 14.4 Hz, 1 H), 6.65 (dd, *J* = 8.8 Hz, *J* = 4.4 Hz, 2 H), 7.05 (q, *J* = 6 Hz, 1 H), 7.24–7.46 (m, 6 H), 7.52–7.66 (m, 5 H), 8.26 (d, *J* = 7.2 Hz, 1 H), 8.84 (s, 1 H), 11,69 (br, 1 H). ^13^C NMR (DMSO-d_6_, 100 MHz): −1.5, 16.8, 17.0, 26.2, 31.6, 34.1, 35.0, 46.0, 52.4, 54.8, 62.4, 64.6, 108.0, 111.2, 114.3, 121.3, 121.4, 122.4, 125.2, 126.8, 127.7, 128.0, 128.2, 128.9, 129.0, 129.5, 130.0, 132.3, 132.4, 148.3, 149.2, 150.7, 151.4, 152.1, 155.0, 159.5, 166.3, 170.1, 171.3, 172.3.

#### 3.2.13. Compound **16** (Folate-DBCO)

To a solution of compound **15** (100 mg, 0.1 mmol) in DMSO (1 mL), TBAF [1.14 mL of 1 M in anhydrous THF, 10 equivalent (eq.)] was added and then stirred at RT. After 10 h stirring, AcOH (1.25 mL) was added and the mixture was poured into a mixture of CHCl_3_ and ethyl acetate (4:1, 25mL). The yellowish precipitate formed in the solution was collected by filtration and then recrystallized in a mixture EtOH:MeOH to give the yellow solid compound **16** (folate-DBCO). ^1^H NMR data is displayed in Figure S2A. ^1^H NMR (DMSO-d_6_, 400 MHz): 1.77–2.04 (m, 5 H), 2.33–2.40 (m, 1 H), 2.88–2.92 (m, 1 H), 3.05–3.11 (m, 1 H), 3.61 (dd, *J* = 14 Hz, *J* = 3.6 Hz, 1 H), 4.10–4.14 (m, 1 H), 4.47 (d, *J* = 6 Hz, 2 H), 5.01 (dd, *J* = 14.4 Hz, *J* = 8.4 Hz, 1 H), 6.63 (dd, *J* = 8.8 Hz, *J* = 2.8 Hz, 2 H), 6.91–6.94 (m, 1 H), 7.07 (br, 1 H), 7.27–7.48 (m, 6 H), 7.55–7.66 (m, 5 H), 7.94 (br, 1 H), 8.63 (s, 1 H). ^13^C NMR data is displayed in Figure S2B. ^13^C NMR (DMSO-d_6_, 100 MHz): 27.4, 31.9, 34.1, 35.0, 46.0, 52.8, 54.8, 108.1, 111.3, 114.3, 121.4, 121.8, 122.5, 125.3, 126.8, 127.7, 128.0, 128.2, 128.7, 129.0, 129.5, 132.4, 148.3, 148.5, 150.6, 151.4, 154.3, 161.5, 165.7, 170.2, 171.7, 174.4. HRMS data is displayed in Figure S2C. HRMS (QSTAR Elite, AB SCIEX, Framingham, MA, USA) calculated for C_37_H_33_N_9_NaO_6_ [M + Na]^+^ 722.2446; obtained [M + Na]^+^ 722.2445.

### 3.3. Click Reaction of Folate-Propargyl or Folate-DBCO with AzPhe-Fmoc

Reaction schemes for the click reactions of folate-propargyl and folate-DBCO with AzPhe-Fmoc are shown in Figure 2. A 1 mM stock of folate-propargyl and a 10 mM stock of BimH_3_ were prepared in DMSO for CuAAC. A 10 mM stock of AzPhe, sodium ascorbate, 2 mM stock of copper (II) sulfate (CuSO_4_) and copper (I) chloride (CuCl) were prepared in H_2_O. 

A 10 mM folate-DBCO stock was prepared in DMF and a 10 mM stock of AzPhe-Fmoc was dissolved in H_2_O for SPAAC. Several trials were performed for both compounds listed in Table 1. RP-HPLC using an Inertsil ODS-3 column (Nacalai tesque Inc., Kyoto, Japan) at 25 °C for 55 min was performed with H_2_O containing 0.1 % (*v*/*v*) TFA (solvent A) and acetonitrile containing 0.1 % (*v*/*v*) TFA (solvent B) as a solvent system with a gradient from 0–0.10 min at 90% A, 5–40 min at 90–50% A, 40–45 min at 50–0% A and 45–47 min at 0–90% A, and flow rate of 1 mL/min. In some cases, a gradient from 0–10 min at 90% A, 5–40 min at 90–30% A, 40–43 min at 30–0% A and 43–45 min at 0–90% A was used, and a flow rate of 1 mL/min.

### 3.4. Synthesis and Purification of Peptides with N-terminal Biotin-PEG_24_

The folate-peptide conjugates were synthesized by conventional Fmoc based solid-phase synthesis methods using a high purity single peptide synthesizer MultiPep CF and micro-column (INTAVIS Co. Ltd., Cologne, Germany). During synthesis, coupling and deprotection steps were carried out in the peptide synthesizer. All peptides were synthesized at the 10 μmol scale. Peptide synthesis is as follows:

Preloaded 0.21 mmol/g of fmoc-Gln(Trt)-NovaSyn TGA (Novabiochem, Darmstadt, Germany), 0.19 mmol/g of fmoc-Ala-NovaSyn TGA (Novabiochem) or 0.24 mmol/g of fmoc-Tyr (tBu)-NovaSyn (Novabiochem) was used for synthesis of GF[AzPhe]IQ, SE[AzPhe]KA and DSE[AzPhe]KAY, respectively. Fmoc deprotection was performed by using 20% (*v*/*v*) piperidine in *N*-methyl-2-pyrrolidone (NMP) or 1% (*v*/*v*) formic acid + 20% (*v*/*v*) piperidine in NMP, depending on the amino acid content of the peptide. For the coupling step, the corresponding amino acid (5 times mol with respect to resin) was incubated with the resin for 30 min in the presence of NMP (8 μL), 0.5 M (2-(1H-benzotriazol-1-yl)-1,1,3,3-tetramethyluronium hexafluorophosphate (150 μL) and 4 M N-methylmorpholine (45 μL). AzPhe (65 ng) was used during each synthesis. After confirming the mass of the synthesized peptides by MALDI-TOF MS, the beads were incubated overnight on a shaker with a mixture of biotin-PEG_24_-NHS (34 mg, 1.5 mol eq.), hydroxybenzotriazole (8.3 mg, 0.6 mol eq.) with the addition of NMP (300 μL). The reaction scheme for *N*-terminal peptide modification with biotin-PEG_24_ modification is shown in Figure 3A. After confirming the mass of the product with MALDI-TOF MS, the peptides were cleaved from the resin using a cleavage cocktail [95.0% (*v*/*v*) TFA, 2.5% (*v*/*v*) triisopropylsilane and 2.5% (*v*/*v*) H_2_O]. Depending on the amino acid content of each peptide, resins were incubated with the cleavage cocktail for 2–4 h in a light protected container with intermittent shaking. The cleavage mixture was then filtered to remove the beads and peptides were precipitated using cold Et_2_O. The resulting precipitate was centrifuged and washed three times with Et_2_O. Et_2_O was removed by overnight vacuum lyophilization and peptides were obtained in powder form. The products were further purified by RP-HPLC using the Inertsil ODS-3 column at 25 °C for GF**Z**IQ and SE**Z**KA: 50 min with a gradient of 1–51% (*v*/*v*) acetonitrile in water containing 0.1% (*v*/*v*) TFA. An Inert Sustain C18 column (Nacalai tesque Inc.) at 50 °C was used to further purify the DSE**Z**KAY peptide. The gradient was 25–55% (*v*/*v*) acetonitrile in water containing 0.1 % (*v*/*v*) TFA for 30 min. Peptides were purified as shown in Figure S3 and analyzed by MALDI-TOF MS (Figure S4A–C) and the results are summarized in Table S1. The purified peptides were lyophilized and stored until required. 

### 3.5. SPAAC Click Chemistry to Conjugate Folate into Peptides

Folate-DBCO (3.55 mg) was dissolved in 1 mM DMSO (2.5 mL). Folate-DBCO was then diluted to 0.1 mM with MeOH [total DMSO = 10% (*v*/*v*)]. A 1 mM stock of ~50% purified peptide-PEG_24_-biotin was prepared in MeOH. 1 mol eq., 0.1 mM (1 mL) biotin-PEG_24_-peptide was mixed with 2 mol eq., 0.1 mM (2 mL) folate-DBCO in 7.14% (*v*/*v*) DMSO and 92.86% MeOH [40]. The mixture was constantly rotated at 5 rpm and 25 °C on a rotator for 16 h under dark conditions. After the reaction, mixtures were purified by RP-HPLC using the Inertsil ODS-3 column at 25 °C for 50 min with a gradient of 1–51% (*v*/*v*) acetonitrile in water containing 0.1% (*v*/*v*) TFA. Folate-peptide conjugates were analyzed by MALDI-TOF MS analysis.

### 3.6. Purification and Refolding of FRα

All steps performed for purification and refolding of FRα were carried out according to our previous report [41]. However, some different reagents were used. For cell body washing, we used 4 M urea instead of Triton X-114. The inclusion bodies were solubilized and purified with 8 M urea instead of 6 M guanidine HCl. Purification and refolding data are shown in Figure S5, and Tables S2 and S3.

### 3.7. BLI Measurements

The binding affinity of refolded FRα toward folate and folate conjugated peptide aptamers was measured by biolayer interferometry at 25 °C using a BLItz system (ForteBio) with kinetics buffer [10 mM PBS, pH 7.4, 0.5% (*w*/*v*) BSA and 0.01% (*v*/*v*) Tween 20]. The measurement procedure has been reported previously [41]. Streptavidin-coated biosensors (SA sensors were hydrolyzed for 2 h in 250 μL kinetics buffer and then soaked with 250 μL folate-PEG_8_-biotin (2.5 μM), or a variety of concentrations of 250 μL folate-peptide-PEG_24_-biotin conjugates at a stirring speed of 2200 rpm. Two baselines were measured for each sensor in kinetics buffer for 30 and 300 s prior to the immobilization and association step, respectively. Folate-PEG_8_-biotin or folate-peptide-PEG_24_-biotin conjugates immobilized to SA biosensors were dipped into FRα solutions for the association step. Dissociation was monitored in 250 μL immune assay kinetics buffer. To eliminate errors from non-specific binding of the analyte (FRα) on the SA biosensor chips, reference data with the same concentrations of analyte were also measured. 

The obtained binding data were analyzed using a 1:1 local analysis mode applied with association and dissociation step corrections by the BLItz Pro1.2 software (ForteBio). The reference measurements were subtracted during data analysis to determine *k*_a_, *k*_d_ and *K*_D_. 

## 4. Conclusions

By conjugation with peptides the affinities of folate to the receptor were enhanced. The conjugation with designed peptides will be useful for enhancement of ligands affinities through the increase of binding sites.

## Figures and Tables

**Table 1:** Reaction conditions of folate-propargyl or folate-DBCO with AzPhe-Fmoc.

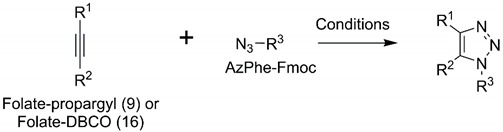			
Folate-alkyne ^a^	Molar Ratio of Folate-alkyne: Azide	Reaction Conditions	Yield (%) ^b^
9	1:1	CuCl (0.1 mM), BimH_3_ (0.1 mM), Na ascorbate (0.1 mM), 11% (*v*/*v*) DMSO + 89% (*v*/*v*) H_2_O, room temperature (RT), 12 h	N.D.
9	1:1	CuCl (0.2 mM), BimH_3_ (0.1 mM), Na ascorbate (0.2 mM), 11% (*v*/*v*) DMSO + 89% (*v*/*v*) H_2_O, 50 °C, 10 h	N.D.
9	1:1	CuSO_4_ (0.1mM), BimH_3_ (0.1 mM), Na ascorbate (0.6 mM), 11% (*v*/*v*) DMSO + 89% (*v*/*v*) H_2_O, MW ^c^, 1h	N.D.
16	1:1	10% (*v*/*v*) DMF + 10% (*v*/*v*) H_2_O + 80% (*v*/*v*) MeOH, RT, 16 h	60
16	1:1	10% (*v*/*v*) DMF + 10% (*v*/*v*) H_2_O + 80% (*v*/*v*) MeOH, 50 °C, 16 h	56
16	2:1	20% (*v*/*v*) DMF + 10% (*v*/*v*) H_2_O + 70% (*v*/*v*) MeOH, RT, 16 h	88

^a^ The number corresponds the compound number in Figure 6. ^b^ High performance liquid chromatography (HPLC) yields; ^c^ Microwave conditions; N.D. Not detected.

**Table 2:** BLI results for the binding affinity of folate and folate-peptide conjugates.

Ligands	*K*_D_ (nM)	*k*_a_ (M^−1^ s^−1^)	*k*_d_ (s^−1^)
Folate	1.14	6.74 × 10^6^	7.69 × 10^−3^
GF**Z**IQ	0.18	4.11 × 10^5^	7.53 × 10^−5^
SE**Z**KA	0.90	8.91 × 10^4^	8.01 × 10^−5^
DSE**Z**KAY	0.24	1.10 × 10^6^	2.65 × 10^−4^

**Z** = folate-conjugated AzPhe.

## Supplementary Materials

Supplementary materials can be found at https://www.mdpi.com/1422-0067/20/9/2152/s1.

## Abbreviations

AcOH	Acetic acid
ADC	Antibody-drug conjugate
AzPhe	4-Azido phenylalanine
BimH_3_	Tris-(2-benzimidazolylmethyl) amine
BLI	Biolayer interferometry
BSA	Bovine serum albumin
brs	Broad singlet
CDI	1,1′-carbonyldiimidazole
CuAAC	Cu(I)-catalyzed alkyne-azide cycloaddition
d	Duplet
DBCO	Dibenzylcyclooctyne
DMF	*N,N*-Dimethylformamide
DMSO	Dimethyl sulfoxide
EDC	1-Ethyl-3-(3-dimethylaminopropyl) carbodiimide hydrochloride
eq	Equivalent
EtOH	Ethanol
Et_2_O	Diethyl ether
Et_3_N	Triethylamine
Fmoc	9-Fluorenylmethyloxycarbonyl group
FR	Folate receptor
FRα	Folate receptor alpha
*k_a_*	Association rate constant
*K_D_*	Equilibrium dissociation constant
*k_d_*	Dissociation rate constant
m	Multiplet
MALDI-TOF MS	Matrix assisted laser desorption/ionization-time of flight mass spectrometry
MeOH	Methanol
MTBD	7-Methyl-1,5,7-triazabicyclo [4.4.0] dec-5-ene
NHS	*N*-hydroxysuccinimide
NMP	*N*-methyl-2-pyrrolidone
PBS	Phosphate buffered saline
PEG	Polyethylene glycol
q	Quartet
RT	Room temperature
s	Singlet
SA	Streptavidin
SPAAC	Strain-promoted alkyne-azide cycloaddition
TBAF	Tetrabutylammonium fluoride
TFA	Trifluoroacetic acid
THF	Tetrahydrofuran
TMS	Tetramethylsilane
