# Supplementary material for: Enhancement of Binding Affinity of Folate to Its Receptor by Peptide Conjugation
Source: Int J Mol Sci. 2019 Apr 30;20(9):2152. doi: 10.3390/ijms20092152 (PMC6539678; doi:10.3390/ijms20092152)
Supplement: Supplementary file 1 [file ijms-20-02152-s001.pdf]

Enhancement of Binding Affinity of Folate to Its Receptor by Peptide Conjugation

Roopa Dharmatti [1,2], Hideyuki Miyatake [1,*], Avanashiappan Nandakumar [3], Motoki Ueda [1,3], Kenya Kobayashi [1], Daisuke Kiga [2,4], Masayuki Yamamura [2] and Yoshihiro Ito [1,2,3,*]

1 Nano Medical Engineering Laboratory, RIKEN Cluster for Pioneering Research, 2-1 Hirosawa, Wako, Saitama 351-0198, Japan; roopa.dharmatti@riken.jp (R.D.), motoki.ueda@riken.jp (M.U.), kenya.kobayashi@alpsalpine.com (K.K.)

2 Department of Computer Science, School of Computing, Tokyo Institute of Technology, 4259 Nagatsuta-cho, Midori-ku, Yokohama 226-8503, Japan, my@c.titech.ac.jp (M.Y.)

3 Emergent Bioengineering Materials Research Team, RIKEN Center for Emergent Matter Science, 2-1 Hirosawa, Wako, Saitama 351-0198, Japan; nandakumar.avanashiappan@riken.jp (A. N.)

4 Department of Electrical Engineering and Bioscience, Waseda University, 2-2 Wakamatsu Cho, Shinjyuku-ku, Tokyo,162-8480, Japan; kiga@waseda.jp (D.K.)

* Correspondence: miyatake@riken.jp (H.M.), y-ito@riken.jp (Y.I.); Tel. : +81-48-467-4979 (Y. I.), Fax: + 81-48-467-9300 (Y. I.)

1. Synthesis of folate derivatives (compounds 9 and 16)

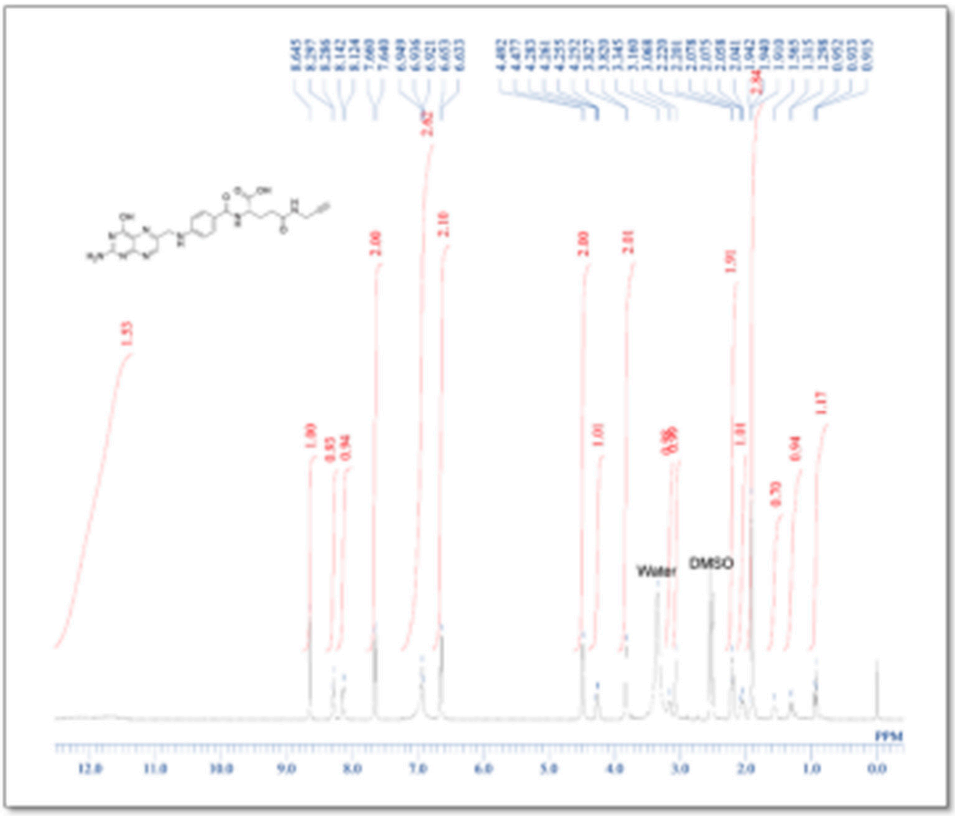

Figure S1. A ^{1}H NMR spectrum of folate-propargyl (compound 9).

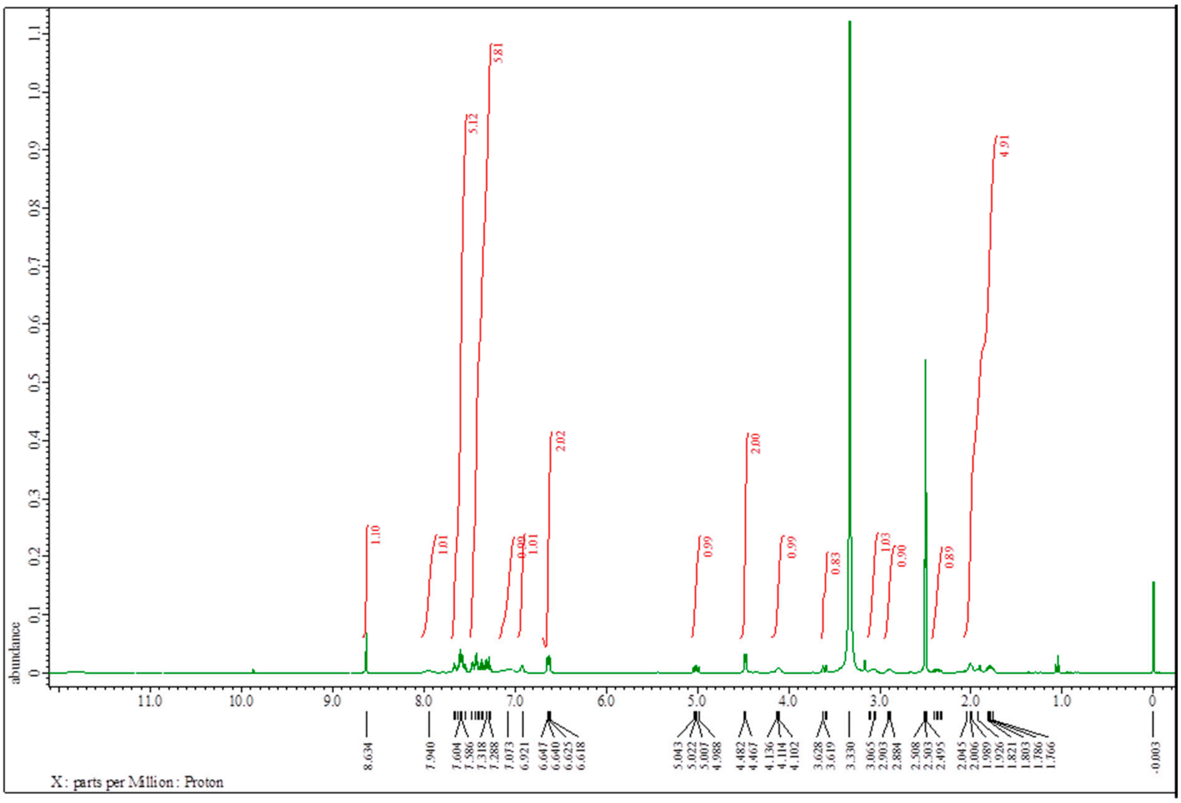

Figure S2A. A ^{1}H NMR spectrum of folate-DBCO (compound 16).

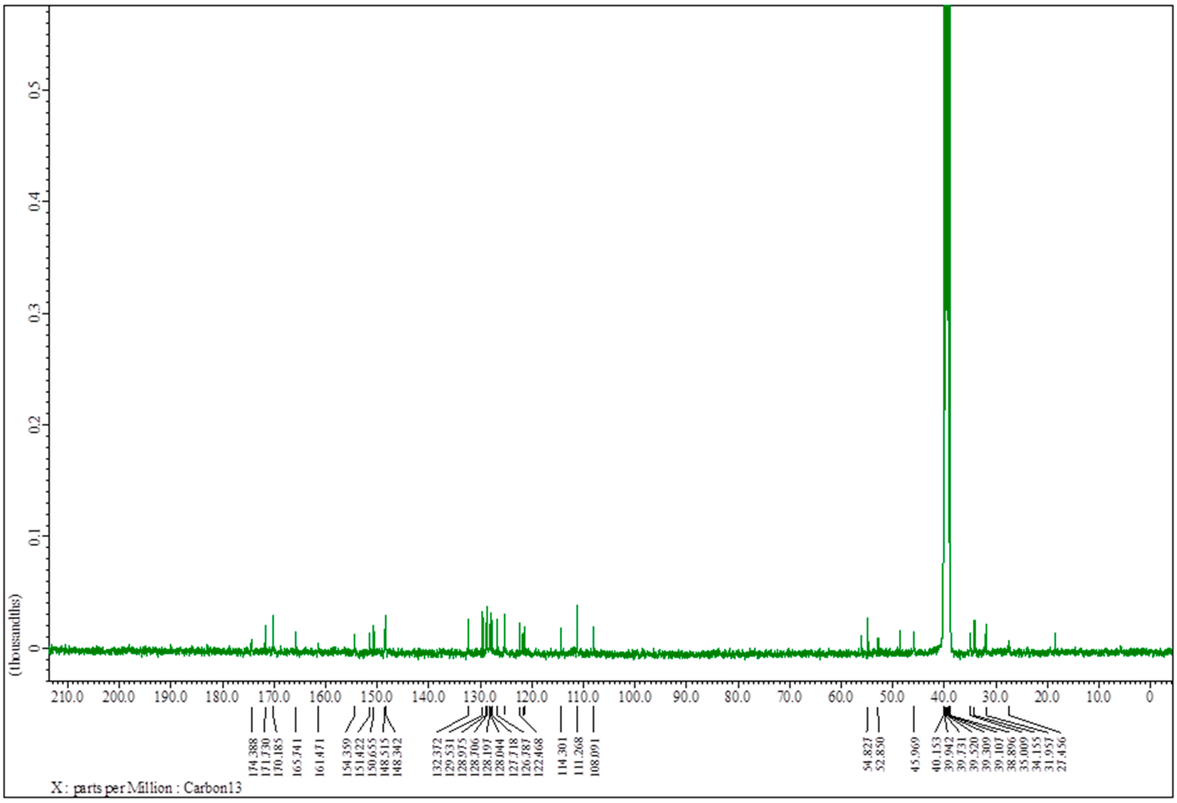

Figure S2B. A ^{13}C NMR spectrum of folate-DBCO (compound 16).

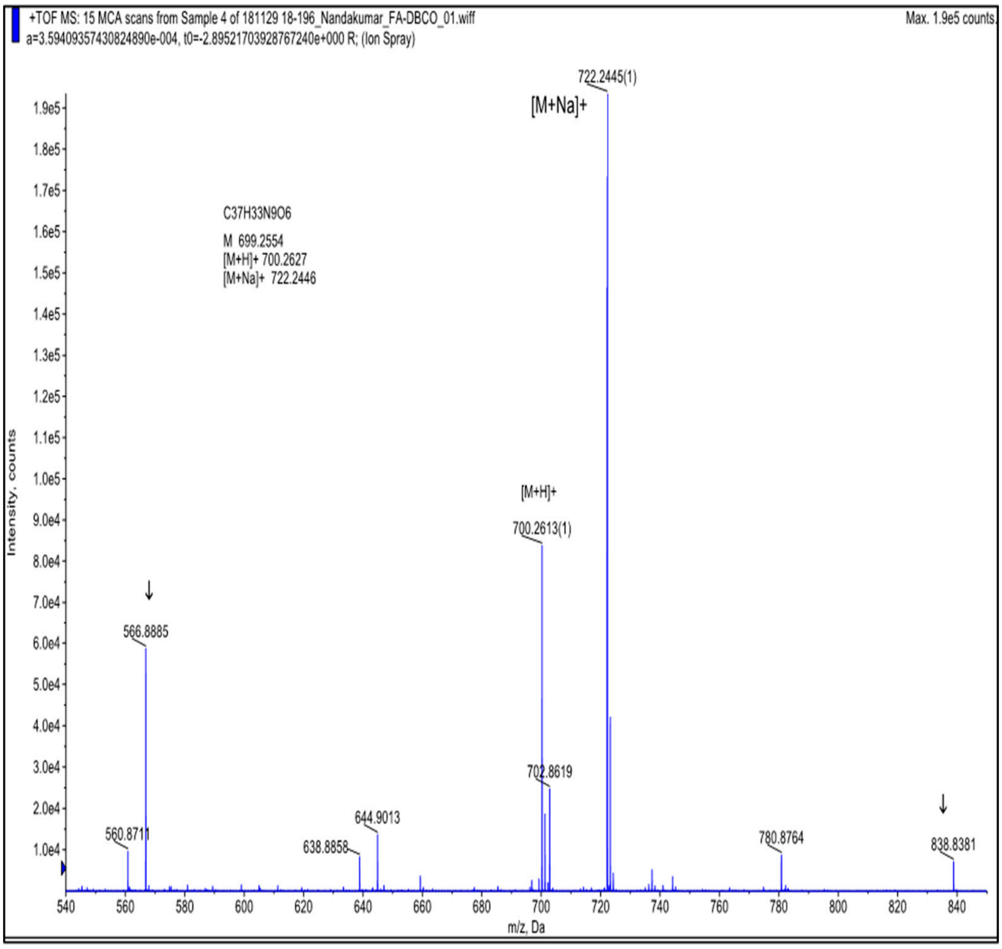

Figure S2C. HRMS of compound 16 (folate-DBCO).

2. Purification of peptides with the N-terminal modification by biotin-PEG$_{24}$ and after folate conjugation by SPAAC.

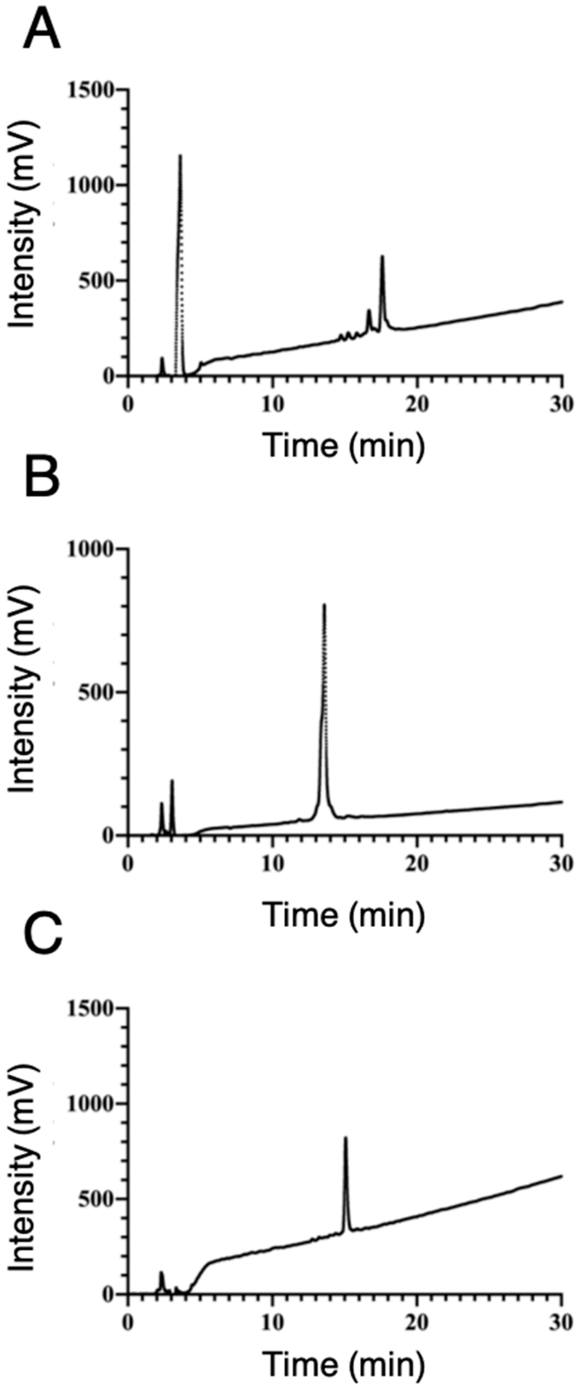

Figure S3. HPLC elution pattern of (A) GF**Z**IQ, (B) SE**Z**KA and (C) DSE**Z**KAY.

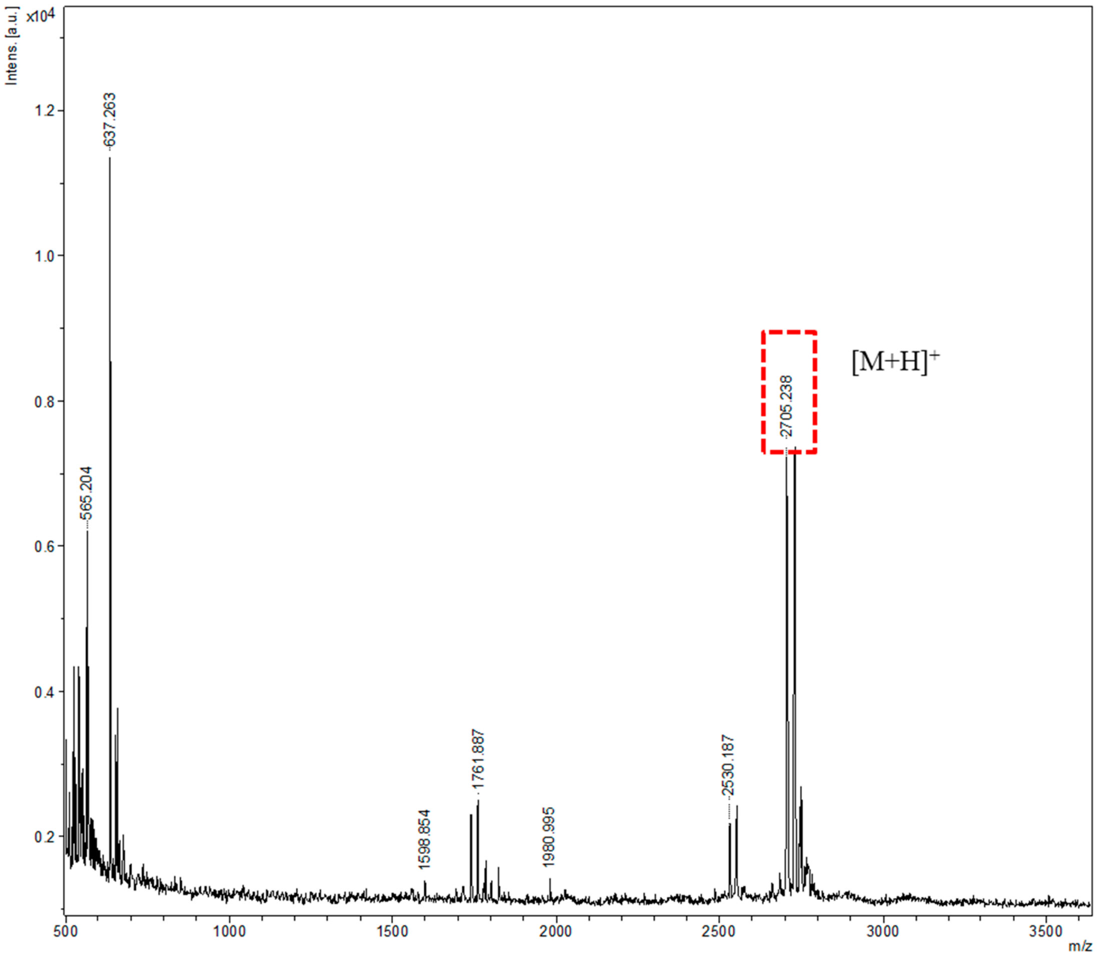

Figure S4A. MALDI-TOF MS analysis of GF**Z**IQ.

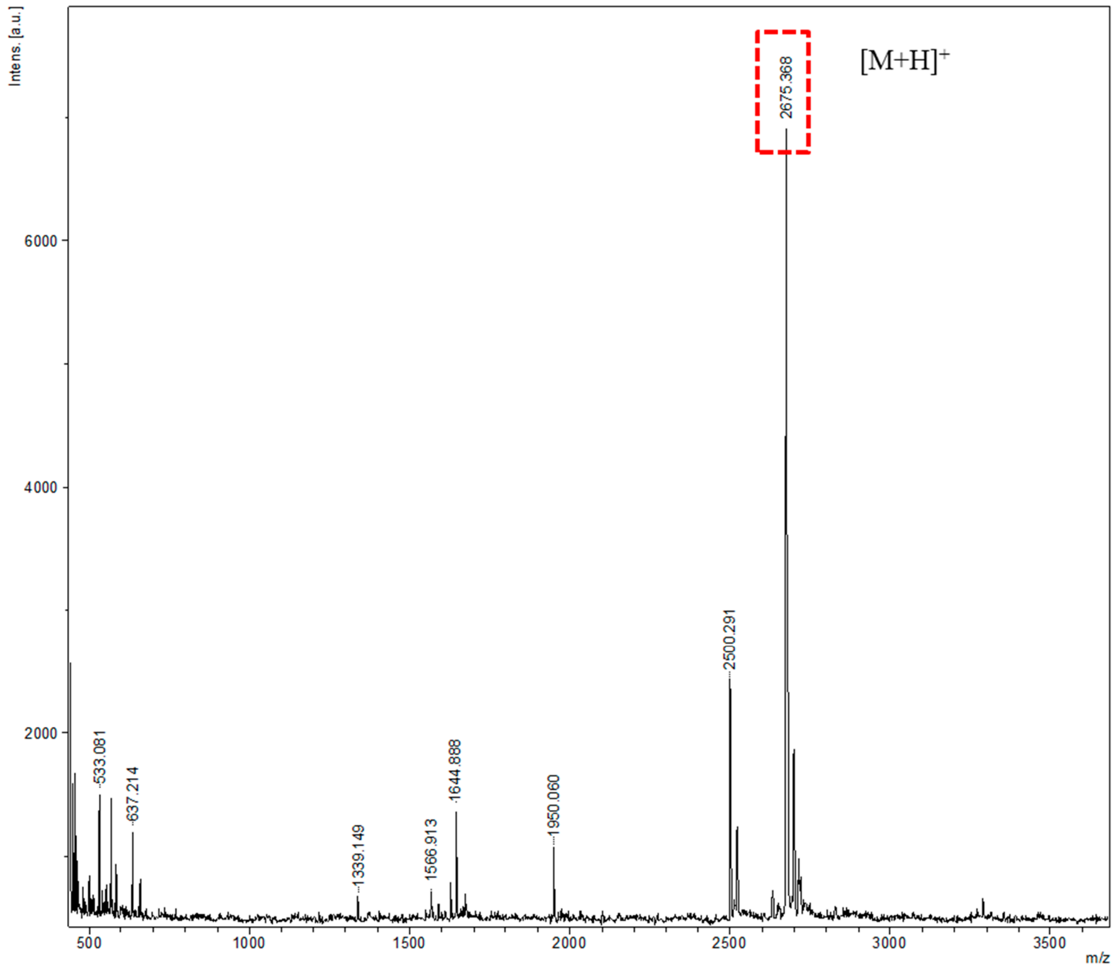

Figure S4B. MALDI-TOF MS analysis of SE**Z**KA.

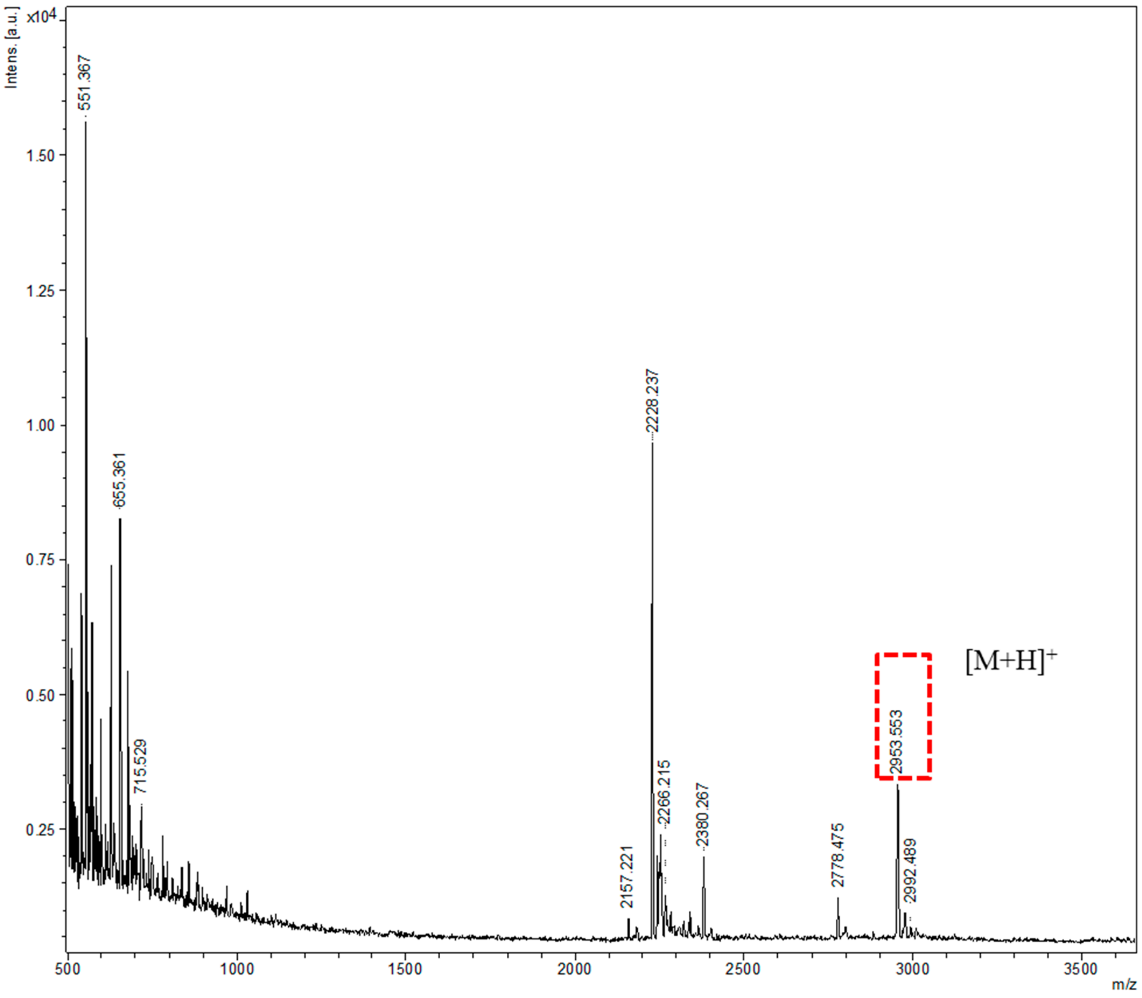

Figure S4C. MALDI-TOF MS analysis of DSE**Z**KAY.

Table S1. MALDI-TOF MS results.

**Peptide Sequences[a]**	**Molecular Mass (Da)**	
	**Calculated [M+H]$^+$**	**Observed [M+H]$^+$**
GF**Z**IQ	2705.3	2705.2
SE**Z**KA	2675.3	2675.4
DSE**Z**KAY	2953.4	2953.6

[a]**Z** = folate conjugated AzPhe.

3. Purification and refolding of FRα.

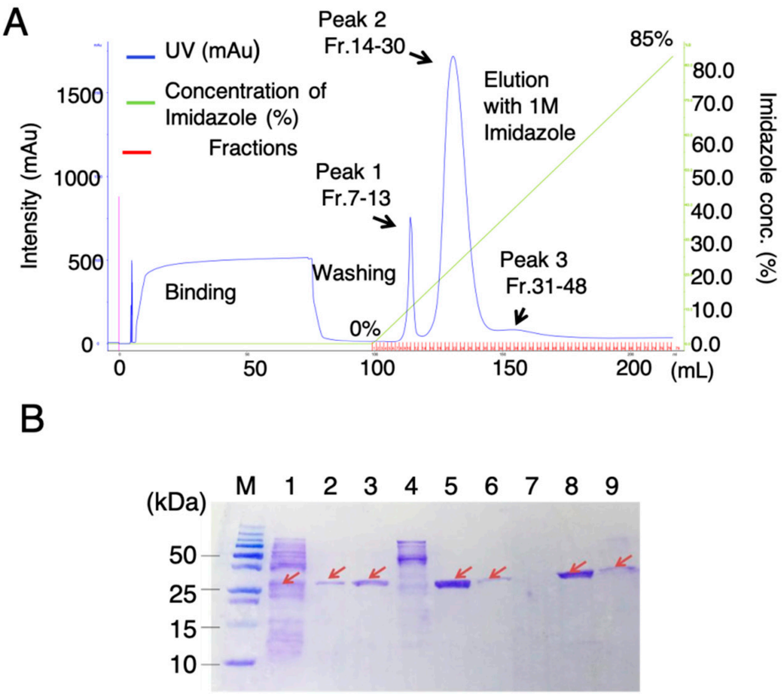

Figure S5. Purification of FRα. (A) FPLC chromatogram of FRα recorded at 280 nm during the immobilized metal affinity chromatography (IMAC) purification procedure. (B) Quality analysis of FRα during each step of purification by 20% SDS-PAGE: M: Molecular mass marker; Lane 1: supernatant; Lane 2: pellet; Lane 3: solubilized inclusion body; Lane 4: FPLC Peak 1 (fractions 7–13); Lane 5 FPLC Peak 2 (fractions 14–30); Lane 6: FPLC Peak 3 (fractions 31–48); Lane 7: FRα in refolding buffer; Lane 8: refolded FRα; Lane 9: precipitate during refolding.

Table S2. Purification steps and buffers.

Steps	Solution contents
Washing of inclusion body	• Sonication buffer: 50 mM Tris-HCl, 100 mM NaCl, 1 mM EDTA, ,4 M urea, pH 7.5, 0.2 mg/mL lysozyme, 0.8 mg/mL DNase I<br>• Washing buffer A: 50 mM Tris-HCl, 100 mM NaCl, 1 mM EDTA, 4 M urea, pH 7.5<br>• Washing buffer B: 50 mM Tris-HCl, 100 mM NaCl, 1 mM EDTA, pH 7.5
Solubilization of inclusion body proteins	• Solubilization buffer: 50 mM Tris-HCl, 8 M urea, 100 mM NaH_2PO_4, pH 8.0, 10 mM 2-mercaptoethanol

Steps	Solution contents
IMAC purification	• Binding buffer:<br>50 mM Tris-HCl, 8 M urea, 100 mM NaH_2PO_4, pH 8.0, 5 mM 2-mercaptoethanol<br><br>• Elution buffer:<br>50 mM Tris-HCl, 8 M urea, 100 mM NaH_2PO_4, 1 M imidazole, pH 8.0, 5 mM 2-mercaptoethanol
Infinite dilution for refolding	• Refolding buffer:<br>50 mM Tris-HCl, 200 mM NaCl, pH 8.0, 1 mM EDTA, 1 mM GSSG, 5 mM GSH, 1 M L-arginine, 10% (v/v) glycerol, pH 8.0
Final concentration	• Replacing buffer:<br>50 mM Tris-HCl, 10% (v/v) glycerol, pH 8.0

Table S3. Summary of the yields of individual steps in the solubilization, purification and refolding of recombinant FRα from inclusion bodies per 10g of *E. coli.*

Steps	FRα (mL)	FRα[a] (mg/mL)	FRα (mg)	FRα (%)
Solubilization of inclusion bodies	80	1.93	154.22	100
**Purification by IMAC**				
Elute peak 1	10.5	0.31	3.27	2.12
Elute peak 2	25.5	1.33	33.99	22.04
Elute peak 3	27	0.11	2.93	1.90
Refolded protein	1050	0.04	44.2	28.66
Soluble protein recovered	0.1	3.58	0.358	0.23

[a]The FRα concentration was determined by measuring the absorbance at 280 nm and using the molar extinction coefficient of 78520 M^{-1} cm^{-1}.
